# Functional Profile of Human Cytomegalovirus Genes and Their Associated Diseases: A Review

**DOI:** 10.3389/fmicb.2020.02104

**Published:** 2020-09-04

**Authors:** Lele Ye, Yunyun Qian, Weijie Yu, Gangqiang Guo, Hong Wang, Xiangyang Xue

**Affiliations:** ^1^Department of Gynecologic Oncology, Women’s Hospital, School of Medicine, Zhejiang University, Hangzhou, China; ^2^Department of Microbiology and Immunology, Institute of Molecular Virology and Immunology, Institute of Tropical Medicine, School of Basic Medical Sciences, Wenzhou Medical University, Wenzhou, China; ^3^First Clinical College, Wenzhou Medical University, Wenzhou, China

**Keywords:** human cytomegalovirus, functional profiling, therapeutic targets, viral genome, viral replication, anti-viral agents

## Abstract

The human cytomegalovirus (HCMV), whose genome is 235 ± 1.9 kbp long, is a common herpesvirus. However, the functions of many of its genes are still unknown. HCMV is closely associated with various human diseases and infects 60–90% of the global population. It can infect various human cells, including fibroblasts, epithelial cells, endothelial cells, smooth muscle cells, and monocytes. Although HCMV infection is generally asymptomatic and causes subtle clinical symptoms, it can generate a robust immune response and establish a latent infection in immunocompromised individuals, including those with AIDS, transplant recipients, and developing fetuses. Currently available antivirals approved for the treatment of HCMV-associated diseases are limited by dose-limiting toxicity and the emergence of resistance; however, vaccines and immunoglobulins are unavailable. In this review, we have summarized the recent literature on 43 newly identified *HCMV* genes. We have described their novel functions on the viral replication cycle, latency, and host immune evasion. Further, we have discussed HCMV-associated diseases and current therapeutic targets. Our review may provide a foundational basis for studies aiming to prevent and develop targeted therapies for HCMV-associated diseases.

## Introduction

The human cytomegalovirus (HCMV), whose linear double-stranded DNA genome is 235 ± 1.9 kbp in length, has the largest genome among human herpesviruses. It has an E-type genome structure consisting of two large reverse domains called long (L) and short **(S)**, each of which is composed of three regions: central unique, unique long, and unique short (Van Damme and Van Loock, 2014). Each region is flanked by terminal (TRL and TRS) and internal (IRL and IRS) inverted repeats (Murphy and Shenk, 2008). The HCMV genome contains various gene families, including RL11, UL14, UL18, UL25, UL82, UL120, US6, US7, US12, and US22. A study has recently demonstrated that the HCMV genome has more than 751 translated open reading frames (ORFs) (Stern-Ginossar et al., 2012; Patro, 2019). Of these, 282 viral transcripts are translationally active, 206 of which possess unique coding potential (Balazs et al., 2017) and one-third of the viral poly-A RNA transcripts are protein-coding (Gatherer et al., 2011). In addition, HCMV encodes four major long non-coding RNAs (lncRNAs) (RNA1.2, RNA2.7, RNA4.9, and RNA5.0) (Tai-Schmiedel et al., 2020) and at least 16 pre-miRNAs and 26 mature miRNAs (Pavelin et al., 2013; Ding et al., 2017; Zhang et al., 2020). Although the functions of many genes are still unknown, the roles and functions of most *HCMV* genes in infective stages have been identified.

HCMV infects 60–90% of the population worldwide (Stevenson et al., 2014) and can infect various human cells, including fibroblasts, epithelial cells, endothelial cells, smooth muscle cells, and monocytes (Gerna et al., 2019). In fibroblasts, HCMV produces abundant progeny (viral particles) through extensive replication and proliferation (Kalejta, 2008b). HCMV-infected monocytes carry viral particles to multiple organs via the bloodstream, thus contributing to productive and persistent infection (Smith et al., 2004, 2007; Nogalski et al., 2011; Chan et al., 2012; Stevenson et al., 2014). After induction, the HCMV-infected monocytes can differentiate into macrophages and further support the replication and expression of viral genes (Stevenson et al., 2014). Further, the inactivation of some US12 family members (e.g., *US18* and *US20*) plays a role in cell tropism by mediating viral replication in specific cells (Cavaletto et al., 2015). The deletion of the *US18* abrogates replication in human gingival tissues (Gurczynski et al., 2014), whereas the *US20* affects the replication of HCMV clinical strains in endothelial cells (Cavaletto et al., 2015).

Primary HCMV infection is generally asymptomatic and causes subtle clinical symptoms in healthy individuals (Gandhi and Khanna, 2004; Reeves and Sinclair, 2008). However, in immunocompromised individuals, it can generate a robust immune response (Magro et al., 2007) and establish a latent infection via immune escape mechanisms. It can be activated from the latent state, which shows a dynamic change of “infection-latency-activation” in immunocompromimsed individuals (Reyda et al., 2014) and permits the life-long persistence of the virus. In productive infection, HCMV exhibits a temporal cascade of gene expression, and these genes can be classified into three, namely *immediate early (IE), early (E)*, and *late (L)* genes (Reeves, 2011; Isomura and Stinski, 2013; Supplementary Table S1). The *major IE* genes (*MIE*), *UL122* and *UL123*, play critical roles in subsequent viral gene expression and the efficiency of viral replication (Isomura and Stinski, 2013).

Human cytomegalovirus is closely associated with various diseases, such as tumors, circulatory system diseases, digestive system diseases, and ophthalmic diseases (idiopathic) (Samanta et al., 2003; Lindholt and Shi, 2006; Magro et al., 2007; DiMaggio et al., 2009; Chen H.P. et al., 2015; Wang H.W. et al., 2016; Wang Z. et al., 2016; Dennis et al., 2018; Guo et al., 2018; Moussawi et al., 2018; Song et al., 2018; Alston and Dix, 2019; Liu et al., 2020). The roles of *HCMV* genes in the occurrence and development of diseases have been previously described (Schleiss, 2011). For example, HCMV encodes a G protein-coupled receptor US28, which activates pro-migratory signaling and mediates vascular smooth muscle cell migration (Melnychuk et al., 2004; Vomaske et al., 2010). On the one hand, US28 enhances the inflammatory properties of cardiovascular diseases (Söderberg-Nauclér, 2006). On the other hand, US28 promotes the invasiveness of diseased cells in atherosclerotic plaques (Krishna et al., 2018). Antivirals licensed for the treatment of HCMV infection and diseases are limited by dose-limiting toxicity and the emergence of resistance (Sinkó, 2019). Furthermore, relevant vaccines and immunoglobulins have not been developed (Xu and Yuan, 2018). Accordingly, detailed analyses of the relationships between *HCMV* genes and their associated diseases are needed for the development of preventive measures and therapeutic intervention.

The associations of *HCMV* genes with diseases have not yet been reviewed in detail, and the current knowledge on the functions of *HCMV* genes on the basis of a previous report (Van Damme and Van Loock, 2014) has to be updated. Thus, in this review, we summarized the recent literature on *HCMV* gene functions and described the relationship between *HCMV g*enes and their associated diseases in detail. Particularly, we focused on the novel functions of 43 *HCMV* genes, namely *UL7, UL8, UL10, UL21A, UL23, UL25, UL26, UL27, UL31, UL34, UL35, UL37.1, UL38, UL45, UL47, UL48, UL50, UL53, UL56, UL71, UL76, UL77, UL112, UL116, UL130, UL131A, UL133, UL136, UL138, UL141, UL146, UL148, UL148D, US12, US18, US20, US21, US25, US28, US29, US31,US33*, and *RNA4.9*. However, those of 39 genes (*RL1, RL5A, RL8A, RL9A, RL9, UL2, UL3, UL6, UL12, UL13, UL14, UL15, UL15A, UL19, UL21, UL22, UL42, UL59, UL62, UL90, UL120, UL121, UL127, UL129, UL140, UL147A, UL148A, UL148B, UL148C, UL149, US1, US13, US14, IS15, US26, US34A, RNA1.2, RNA5.0*, and *IRL14*) remain unknown, and further studies are needed to elucidate their biological function. Overall, our comprehensive review provides a foundational basis for future research aimed at the prevention and targeted treatment of HCMV-associated diseases.

## Gene Function Annotation Data

Human cytomegalovirus genes are involved in diverse processes, including latency, immunomodulation, assembly, maturation, egress, viral protein, gene expression and regulation, replication, cell tropism and cell type-specific replication, modulation of the host cell cycle and protein synthesis, viral growth, cellular tracking, nucleotide repair and modification, entry, viral spread, manipulation of host cell signaling pathways, apoptosis, angiogenesis, and tumor formation (Van Damme and Van Loock, 2014; Supplementary Table S2).

According to their role in viral growth, *HCMV* genes may be classified as essential or dispensable, and their deletion can cause moderate or severe growth defects or enhanced growth (Schwartz and Stern-Ginossar, 2019). In terms of the virus life cycle, *HCMV* gene functions can be divided into entry, replication, assembly, maturation, and egress. Six genes (*UL35, UL73, UL74, UL75, UL100*, and *UL115*) are involved in cell entry. Following entry, a temporal cascade begins, leading to the expression of *immediate early (IE), early (E)*, and *late (L)* genes. There are 36 genes are necessary for DNA replication, ten of which (*UL26, UL31, UL34, UL37.1, UL133, UL136, UL141, US29, US33*, and *RNA4.9*) are recently identified. *UL36, UL37, UL37.1, UL37.3, UL38, UL61, UL123, UL138, UL148D, US1, US2, US3, TRS1*, and *IRS* are *IE* genes, whereas there are 59 *E* genes including *UL87, UL104, UL112*, and *UL114*. There are 55 *early-late* (*E/L*) genes (such as *UL3, UL40, UL46*, *UL72*) and at least 75 *L* genes, such as *UL2, UL7*, and *UL14*, which are expressed following the onset of viral DNA replication. When a new genome is produced, DNA is packaged in a newly formed capsid. Immature viruses complete the maturation process, and finally, the virus is released. Thirty-five genes are involved in assembly, maturation, and egress, including 8 newly identified genes (*UL35, UL47, UL48, UL50, UL53, UL56, UL71*, and *UL77*). After viral egress, HCMV can now cause various diseases.

We further classified the genes based on their roles in immunomodulation, apoptosis, and angiogenesis/tumor formation. We found 61 genes related to the latent infection state, only two of which (*miR-UL148D* and *miR-US29*) are newly discovered (Meshesha et al., 2016; Pan et al., 2016). *HCMV* genes related to immunomodulation and apoptosis are also determinants of virus survival time. A total of 57 genes are related to immune regulation, whereas 14 are related to apoptosis. Of these, 15 genes (*UL8, UL10, UL23, UL26, UL31, UL45, UL48, UL112, UL130, UL131, UL148, US12, US18, US20*, and *US31*) related to immunomodulation and six genes (*UL37.1, UL38, UL76, UL138, UL148D*, and *US21*) related to apoptosis are newly identified. *UL7, UL112, US28*, and *RNA4.9* are involved in angiogenesis/tumor formation. Lastly, the number of recently discovered genes related to gene expression/regulation, cellular trafficking, nucleotide repair/modification, and virion stability is 29, 8, 5, and 1, respectively.

## Genes Involved in the Viral Life Cycle

**FIGURE 1:**
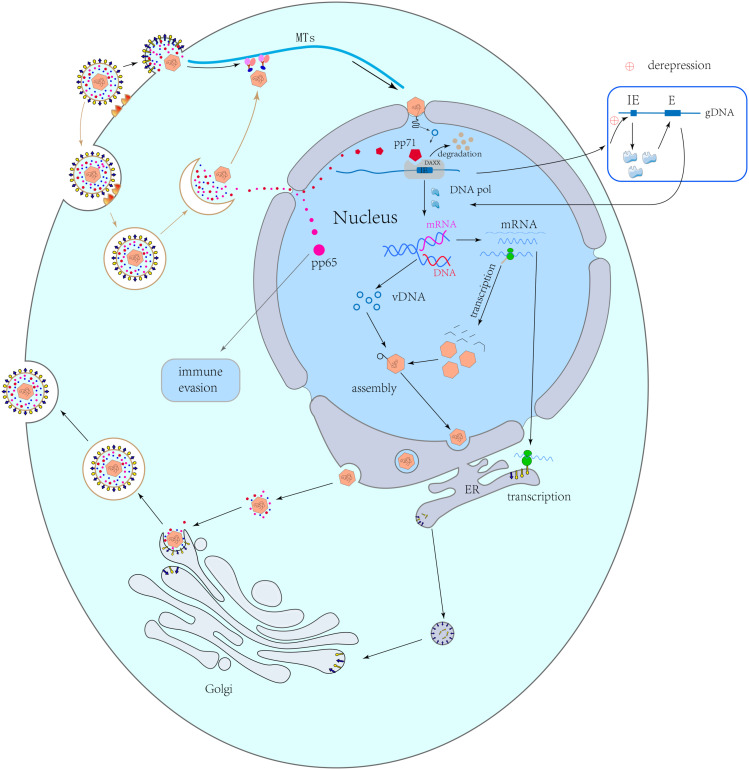
Viral life cycle. Capsid-related proteins transport capsids along the microtubules to the nuclear pores. Capsids remain in the cytoplasm and release DNA into the nucleus. pp71 can be independently transported into the nucleus, where it combines with Daxx to cause its degradation, resulting in the derepression of viral *immediate early (IE)* genes and the activation of *early (E)* genes after transcription and translation. The viral early proteins induce viral genome replication. Following late gene transcription and translation, the viral structural proteins are synthesized and are assembled with the replicated genome to form a new naked virus, which passes through the nuclear membrane via an envelopment-de-envelopment pathway. The naked virus migrates to an assembly site on a Golgi-derived vesicle, where it obtains the final envelope with viral glycoproteins. Finally, virions are released by budding.

Most *HCMV* genes play important roles in its viral life cycle, which involves four stages, namely entry, replication, assembly and maturation, and egress (Figure 1).

### Entry

HCMV infects host cells through two mechanisms, namely membrane fusion and endocytosis, that involve different envelope glycoprotein complexes (Nguyen and Kamil, 2018). The complexes include a trimer complex composed of gH/gL/gO (UL75/UL115/UL74) and a pentamer complex composed of gH/gL/UL128/UL130/UL131 (Huber and Compton, 1998; Wang and Shenk, 2005). Trimers interact with the platelet-derived growth factor receptor α (PDGFRα) and subsequently mediate the pH-independent membrane fusion in fibroblasts (Wu et al., 2017). In contrast, pentamers interact with neuropilin-2 (Nrp2) via low pH-dependent endocytosis in epithelial and endothelial cells (Martinez-Martin et al., 2018; Nguyen and Kamil, 2018). In addition, several reports also found that CD147, CD46, and OR14I1 serve as epithelial entry factors (Nguyen and Kamil, 2018; Vanarsdall et al., 2018; Stein et al., 2019; Xiaofei et al., 2019). The transforming growth factor β receptor type 3 (TGFβRIII) and neuregulin-2 (NRG2) are additional hits for trimers to promote viral entry in fibroblasts (Nguyen and Kamil, 2018; Vanarsdall et al., 2018; Gerna et al., 2019). Viral cell tropism is correlated with the levels of gH/gL/gO and gH/gL/UL128/UL130/UL131 (Zhou et al., 2013). Zhou et al. (2013) found that the levels of gH/gL/gO are higher than those of gH/gL/UL128/UL130/UL131 in TR, TB40/e, AD169, and PH virions, whereas those of gH/gL/UL128/UL130/UL131 are higher in Merlin virions. In epithelial-derived viruses, the ratio of gH/gL/UL128/UL130/UL131 to gH/gL/gO is increased by approximately two-fold (Wang et al., 2007). The surface gM/gN (UL100/UL73) dimer and the gB (UL55) trimer are also involved in viral entry (Gardner and Tortorella, 2016; Foglierini et al., 2019). gM/gN plays an important role in adhesion by interacting with heparin sulfate proteoglycans on the cell surface, whereas the gB trimer acts as a proximal mediator during membrane fusion (Eisenberg et al., 2012; Nguyen and Kamil, 2018).

### Replication and Gene Expression

After passing through the membrane, HCMV transports its capsid along the microtubules to the nuclear pore by hijacking the intracellular machinery (Kalejta, 2008a). In this process, some tegument proteins remain in the cytoplasm or bind tightly to the capsid and mediate the nuclear-pore transport. Other proteins, such as pp65 (pUL83) and pp71 (pUL82), are transported into the nucleus independently (Kalejta, 2008b). The former (pp65) contributes to viral replication, whereas the latter (pp71) mainly affects gene expression (Biolatti et al., 2018a; Kalejta and Albright, 2020). Additionally, the interaction between the pUL47/pUL48 dimer and the capsid can promote the former’s transfer to the nuclear pore complex (Kalejta, 2008a). The capsid is finally dissociated on the microtubule, docks to the nuclear pore, and releases DNA into the nucleus (Kalejta, 2008b). After pp71 entry into the nucleus, it combines with death domain-associated protein (Daxx), which binds to histone deacetylases (HDACs) to repress transcription, thereby causing the degradation of Daxx and inducing the activation of the *IE* genes (Saffert and Kalejta, 2006). Then, as replication begins, the proteins in IE (pIE) can act as activators to promote the expression of *early* and *late* genes via their interaction with cytokines (Adamson and Nevels, 2020). Early proteins are mainly transcription factors and polymerases involved in viral DNA replication, transcription, and protein synthesis, whereas late proteins are mainly structural proteins synthesized after viral DNA replication (Gruffat et al., 2016). A wide range of viral genes are involved in viral replication. For instance, HCMV *UL34* encodes a sequence-specific DNA binding protein (pUL34), which interacts with pUL84, pIE2, and pUL44 and contributes to the establishment of a nuclear environment necessary for viral gene expression and DNA replication (Rana and Biegalke, 2014; Slayton et al., 2018). pUL44 is an obligate nuclear-resident, non-structural viral protein for HCMV DNA replication (Neo et al., 2019). Recent studies have shown that *UL133, UL136, UL141, US29*, and *US33* are also involved in viral DNA replication (Caviness et al., 2014, 2016; Shen et al., 2014; Guo et al., 2015; Zou et al., 2018).

### Assembly, Maturation, and Egress

There are four core components of HCMV capsids, namely major capsid protein (MCP/pUL86), minor capsid binding protein (TRI1/pUL46), minor capsid protein (TRI2/pUL85), and smallest capsid protein (SCP/pUL48A), assembled with the help of pUL80 (Gibson, 2008; Tandon and Mocarski, 2012). During the maturation of virion particles, other capsid-related proteins bind to the capsid. pUL77 and pUL93 stabilize DNA packaging (Köppen-Rung et al., 2016). After capsid maturation, an enzyme complex called a terminase, consisting of pUL51, pUL52, pUL56, pUL77, pUL89, and pUL93, binds to concatemeric viral DNA and cleaves it into unit-length genomes for DNA packaging (Ligat et al., 2018). Neuber et al. (2017) suggested that the mutual interplay between terminase proteins (pUL51, pUL56, and pUL89) can protect the viral or cellular pathways which are vital for the HCMV life cycle from the detrimental effects of incomplete terminase assembly. After assembly, the naked virus leaves the nucleus with the help of the nuclear egress complex (NEC) in an envelopment-deenvelopment-reenvelopment manner (Mettenleiter et al., 2006; Kalejta, 2008b). The core NEC is composed of pUL50 and pUL53, whereas the HCMV-specific multi-component NEC is mainly composed of pUL97, p32/gC1qR, emerin, protein kinase C (PKC), and additional proteins (Milbradt et al., 2018). Milbradt et al. proposed one potential mechanism by which pUL53 and the NEC-related kinase pUL97 bind to the nucleocapsid at the inner nuclear membrane. By complexing with pUL53 and the membrane-anchored pUL50, pUL97 may cause the virus to enter the perinuclear space via phosphorylating the remaining nuclear lamin A/C to disrupt the nuclear lamina (Sharma et al., 2015; Milbradt et al., 2018). Additionally, pUL93 interacts with both components of the NEC (pUL50 and pUL53), suggesting that pUL93 is related to the NEC (DeRussy et al., 2016).

Following nuclear egress, additional tegumentation and final envelopment occur once the capsid is transported to the virion assembly compartment (vAC) in the cytoplasm. This is followed by its excretion from the cell (Das et al., 2007; Das and Pellett, 2011; Alwine, 2012; Hook et al., 2014). The vAC is formed by the reorganization of Golgi bodies via HCMV miRNAs, thus providing additional structural proteins to the nucleocapsid for virion maturation (Alwine, 2012; Hook et al., 2014). pUL48, pUL94, and pUL103 play roles in virion tegumentation by contributing to cytoplasmic vAC biogenesis (Das et al., 2014). Furthermore, pUL48 directs pUL47 to the vAC to promote secondary envelopment and tegumentation (Cappadona et al., 2015). The TB-47stop virus, whose pUL47 expression is blocked, showed severe growth defects in host cells and accumulated non-enveloped capsids in the cytoplasm (Cappadona et al., 2015). Finally, the HCMV life cycle is completed for approximately 72 h (Lee and Grey, 2020).

### Latency

Although most virus can be effectively eliminated upon the activation of host immune system, HCMV can still establish life-long latency once infected. There are 61 genes related to latency, namely *RL2*, *RL4*, *RL6*, *UL4*, *UL5*, *UL17*, *UL28*, *UL30*, *UL32*, *UL37*, *UL38*, *UL39*, *UL40*, *UL44*, *UL50*, *UL52*, *UL61*, *UL64*, *UL65*, *UL67*, *UL68*, *UL70*, *UL73*, *UL75*, *UL76*, *UL79*, *UL80*, *UL81ast/LU NA*, *UL8/pp71*, *UL84*, *UL87*, *UL95*, *UL98*, *UL99/pp28*, *UL105*, *UL108*, *UL110*, *UL111A*, *UL114*, *UL115*, *UL122*, *UL123*, *UL124*, *UL125*, *UL126A*, *UL128*, *UL132*, *UL133*, *UL135*, *UL138*, *UL144*, *UL145*, *UL148D*, *UL150*, *US17*, *US28*, *US29*, *US32*, *US34*, *RNA2.7*, and *RNA4.9*.

The transcript of the UL115.5A region, expressed during latent infection and homologous to interleukin 10 (IL-10), can help the virus escape immune recognition (Jenkins et al., 2004). The *UL111A* and *UL111.5A* genes encode IL-10 that also play a role as immunomodulatory cytokine (Jenkins et al., 2004). IE1 expression can prevent the apoptosis induced by *US28*, which may function as a proapoptotic factor (Jenkins et al., 2004). Furthermore, *US28* causes potential infections by weakening a variety of cellular signal transduction factors, including mitogen-activated protein kinase and nuclear factor kappa-B (NF-κB) (Krishna et al., 2017). *UL138* has long and short isoforms, both of which suppress major IE gene transcription and promote latency (Lee et al., 2016). *Hcmv-miR-UL36* down-regulates HCMV *UL138*, indicating that *hcmv-miR-UL36* may be a new latent period-related determinant of viral miRNAs contributing to HCMV replication (Huang et al., 2013; Zhang et al., 2020). The protein isoforms encoded by *UL136* regulates the latent and replication states of infection, whereas the complex interaction between *UL136* subtypes balances the replication and latency period of HCMV (Caviness et al., 2016). *miR-UL148D* and *US29* are recently discovered loci associated with latency (Lau et al., 2016; Pan et al., 2016). By targeting the host cell’s immediate early response gene 5 (IER5) and inhibiting the activin A-triggered secretion of IL-6, *miR-UL148D* facilitates viral infection during latent infection (Lau et al., 2016; Pan et al., 2016). In addition, the star “passenger” form of *miR-US29* is preferentially expressed during latency, as evidenced by a shift in the abundance of the two arms of *miR-US29* between the productive and latency stages (Meshesha et al., 2016). A recent study has also suggested that *UL76* has a dominant negative effect on replication, which may be related to latency (Wang et al., 2004).

## Immune Evasion

HCMV also encodes abundant genes and proteins closely related to immunoregulation (Gatherer et al., 2011). During its long-term coevolution with the host, HCMV has developed a variety of immune escape strategies. The immune system therefore cannot completely clear HCMV (Söderberg-Nauclér, 2008).

### Innate Response

The innate immune system is the host’s first line of defense against invading pathogens (Akira et al., 2006). When the human body is infected by microbes, pattern recognition receptors (PRRs) detect pathogen-associated molecular patterns (PAMPs) and induce the production of type I interferons (IFNs) and pro-inflammatory cytokines (Medzhitov, 2007). Among the PRRs, the cyclic guanosine monophosphate-adenosine monophosphate (cGAMP) synthase (cGAS) has been shown to act as a DNA sensor that transmits DNA virus infection signals to various cell lines (Medzhitov, 2007; Li et al., 2013). cGAS is capable of recognizing double-stranded DNA and catalyzing the synthesis of cGAMP, which functions as a second messenger in innate inmune (Wu et al., 2013). Subsequently, cGAMP activates the mediator of IRF3 activation (MITA), which triggers innate antiviral response (Zhong et al., 2008). There are several mechanisms by which HCMV antagonizes this signaling pathway. For example, the tegument protein UL82 inhibits the cellular transport and activation of MITA to evade antiviral immunity (Fu et al., 2017). UL31 also mediates immune invasion through the inhibition of DNA sensing by cGAS (Huang et al., 2018). Further, UL83 interacts with cGAS and interferon gamma-inducible protein 16 (IFI16) in the nucleus, thereby inhibiting the induction of IFN production (Biolatti et al., 2018b). The IE1 and IE2 proteins, which are synthesized after HCMV infection, can inhibit the IFN signal transduction and thereby inhibit the innate immune response (Paulus and Nevels, 2009). In addition, it has been suggested that *US10* attenuates IFN-β (Park et al., 2019). The inhibition of cGAS activation and MITA trafficking by UL42 is contributes to the evasion of immune antiviral immunity by HCMV (Fu et al., 2019).

The innate immune system is primarily composed of natural killer (NK) cells, dendritic cells, and macrophages (Takeuchi and Akira, 2010). NK cells are capable of recognizing virus-infected cells and play an important role in the host defense against HCMV (Dell’Oste et al., 2020). *US9* can mediate IFN-β production and block NK-cell activation (Seidel et al., 2015). pUL135 remodels the actin cytoskeleton and weakens the effects of F-actin filaments during synapse formation (Stanton et al., 2014). It can thus weaken the function of cytotoxic immune effector cells and subsequently evade immune recognition in infected cells (Stanton et al., 2014). *Hcmv-miR-UL112* attenuates NK-cell activity by inhibiting type I interferon secretion (Huang et al., 2015). *UL148* increases degranulation in both cytotoxic T lymphocytes and NK cells against HCMV-infected cells, including NK-driven antibody-dependent cellular cytotoxicity (Wang et al., 2018). US12 family genes control immune ligands and suppresses NK-cell activation (Fielding et al., 2017).

### Adaptive Response

Adaptive immunity is vital for controlling primary HCMV infection. After infection, the host immune response can be enhanced by increasing the number of highly differentiated cells, such as CD4+T and CD8+T cells (Manandhar et al., 2019). The US2-US11 region encodes at least five glycoproteins (US2, US3, US6, US10, and US11), which are specifically used to interfere with the expression of the antigen peptides of CD8+T cells and inhibit MHC class I antigen presentation (Kim et al., 2011). In addition, US2 and US11 products are expressed during the early phase of infection and down-regulate class I molecules during viral infections (Hansen and Bouvier, 2009; Gabor et al., 2020). Meanwhile, HCMV *miR-US4-1* inhibits CD8+T cell response by targeting the endoplasmic reticulum aminopeptidase 1, which plays an important role in the production of antigenic peptides and blocks the production of viral epitopes (Kim et al., 2011). HCMV *miR-UL112-5p* also targets the endoplasmic reticulum aminopeptidase 1 to inhibit the processing and presentation of the HCMV pp65_495__–__503_ peptide to specific cytotoxic T lymphocytes (Kim et al., 2011; Huang et al., 2015; Romania et al., 2017). Additional gene products involved in immunomodulation have also been identified. After the infection of THP-1 cells, pUL8 decreases the levels of proinflammatory factors to inhibit inflammation (Perez-Carmona et al., 2018). The interaction of pUL10 with leukocytes mediates immunosuppression by reducing T-cell proliferation and cytokine production (Bruno et al., 2016).

### Intrinsic Response

The mammalian immune system consists of the innate and adaptive immune responses. However, a number of studies have suggested the existence of a special form of innate immunity known as intrinsic response, which forms the third branch of the immune system (Bieniasz, 2004). The intrinsic immune system is composed of a variety of cellular proteins termed restriction factors (RFs) (Bieniasz, 2003). These cellular proteins, including the IFN-γ inducible protein 16, a 100-kDa speckled protein (Sp100), promyelocytic leukemia protein (PML), and the human death domain-associated protein 6 (hDaxx), can interfere with the various stages of the virus replication cycle (Glass and Everett, 2013). Sp100, hDaxx, and PML also form a subnuclear substructure known as nuclear domain 10 (ND10), which can restrict the expression of immediate-early gene and influence the virus replication cycle (Glass and Everett, 2013). However, HCMV is capable of overcoming these RFs by encoding viral proteins, such as pp71, IE1, UL97, and pp65. For instance, UL97 and pp65 (pUL83) antagonize IFI16, resulting in the mislocalization of IFI16 into the cytoplasm (Dell’Oste et al., 2014; Biolatti et al., 2016). IE1 and pp71 can block or reverse the inhibitory effects of ND10 during viral replication through the modification or destruction of nuclear domain 10 proteins (Hofmann et al., 2002; Cantrell and Bresnahan, 2005).

## HCMV-Related Infection and Diseases

**FIGURE 2:**
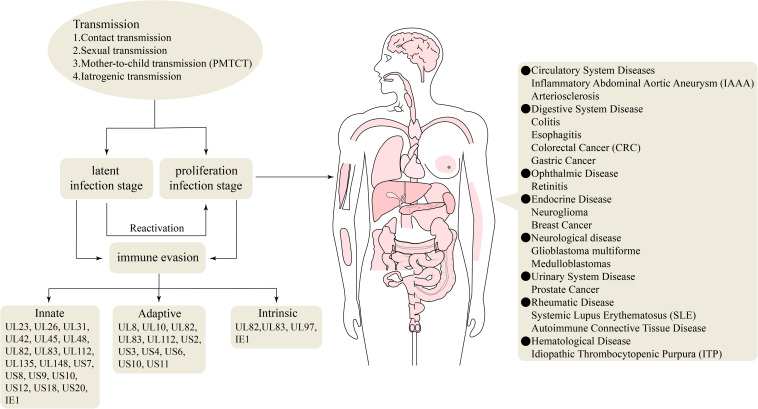
Genes involved in the immune evasion and HCMV-associated diseases.

Humans are the only host of HCMV, and most human organs and tissues can be infected. Upon infection, a long-term HCMV infection can be established, which correlates with chronic inflammation that influences the development of cardiovascular diseases and some types of cancers in otherwise immunocompetent individuals (Söderberg-Nauclér, 2006, 2008; Nogalski et al., 2014). Primary and active infection causes serious diseases in immunotolerant individuals. Active infection can lead to various diseases, such as arteriosclerosis, colitis, esophagitis, colorectal cancer (CRC), gastric cancer (GC), idiopathic thrombocytopenic purpura (ITP), prostate cancer, systemic lupus erythematosus (SLE), systemic sclerosis (SSc), autoimmune connective tissue disease, neuroglioma, breast cancer, and retinitis (Samanta et al., 2003; Magro et al., 2007; DiMaggio et al., 2009; Chen H.P. et al., 2015; Wang H.W. et al., 2016; Wang Z. et al., 2016; Arcangeletti et al., 2018; Dennis et al., 2018; Guo et al., 2018; Moussawi et al., 2018; Song et al., 2018; Alston and Dix, 2019; Liu et al., 2020; Figure 2). In immunocompromised individuals, including those with acquired immunodeficiency syndrome (AIDS), transplant recipients, and developing fetuses, the incidence of HCMV infection and mortality are elevated (Crough and Khanna, 2009; Nogalski et al., 2014).

### Neoplastic Diseases

Although the mechanism of HCMV in many cancers is unclear, some studies have shown that HCMV plays a role in tumor regulation and influences cancer development (Michaelis et al., 2011). Taher et al. (2013, 2014) reported that HCMV has a high prevalence in the brain metastases of patients with primary breast and colorectal cancers, which suggests its involvement in tumor metastasis. Jin et al. (2014) found that *UL135* and *UL136* transcripts are only detected in gastric cancer patients, especially in the tissues of those in stage III. Roman abraheta et al. suggested that HCMV may up-regulate the adhesion of prostate cancer cells to the endothelial cells and extracellular matrix proteins, accompanied by the overexpression of integrin (Blaheta et al., 2006).

In addition, HCMV encodes pUL123 and pUL122 that prevent glioblastoma cell death by promoting angiogenesis, which is vital for tumor growth and survival in glioblastoma multiforme (Herbein, 2018). *US28* induces angiogenesis and activates Gαq-linked signaling pathways, thereby increasing the expression of vascular endothelial growth factors in HCMV-infected glioblastoma cells (Maussang et al., 2009; Heukers et al., 2018). *US28* induces the angiogenic phenotype in patients with colorectal cancer (Maussang et al., 2006). Meanwhile, *US33* activates the proliferation and angiogenesis and inflammatory signaling pathways in glioblastoma cells (Heukers et al., 2018; van Senten et al., 2019). *UL111A* affects the survival time of gastric carcinoma patients by altering the number of infiltrating T cells (Liu et al., 2020).

### Non-neoplastic Diseases

Frequent and active CMV infection plays a vital role in the induction and acceleration of chronic inflammatory reactions in the aortas of patients with inflammatory abdominal aortic aneurysm (Lindholt and Shi, 2006; Liu et al., 2020). By aggravating the apoptosis of endothelial cells induced by oxidized low-density lipoprotein, mi*R-US25-1* may promote atherosclerosis (Fan et al., 2014). Some studies reported that HCMV infection is associated with the pathogenesis of SLE. The titers of anti-HCMV IgG and IgM antibodies have been demonstrated to be significantly higher in SLE patients than in healthy individuals. Furthermore, the positivity rate of HCMV *UL55* was significantly higher in SLE patients than in healthy controls, suggesting that *UL55* may play an important role in the development of SLE (Chen J. et al., 2015). Another study suggested the association between HCMV infection and SLE via the human antibody response targeting UL44 (Neo et al., 2019). *UL44* is redistributed to the cell surface during virus-induced apoptosis as part of a complex with these autoantigens like nucleolin and dsDNA (Neo et al., 2019). Meanwhile, *US31* induces inflammation in mono-macrophages by promoting NF-κB2 activation, which may also contribute to the progression of SLE (Guo et al., 2018). HCMV encodes *UL94* related to SSc, which may function in cellular gene expression and induce apoptosis in human endothelial cells (Lunardi et al., 2000).

## Potential Therapeutic Targets

Available anti-virals for HCMV-related diseases include ganciclovir, foscarnet, and cidofovir (Sinkó, 2019). Ganciclovir acts as a competitive inhibitor of deoxyguanosine (Upadhyayula and Michaels, 2013). Its greater accumulation in CMV-infected cells suggests that ganciclovir has better antiviral activity than acyclovir (Marshall and Koch, 2009). Meanwhile, foscarnet reversibly inhibits viral DNA polymerases (De Clercq and Li, 2016). In CMV retinitis patients administered with induction therapy with foscarnet, ocular symptoms were initially relieved but recurred soon after stopping the treatment (Heiden et al., 2019). Cidofovir competitively inhibits DNA elongation (Britt and Prichard, 2018) and is helpful for treating colitis (Baniak and Kanthan, 2016). Cottone et al. (2001) showed that antiviral treatment can relieve the clinical symptoms of patients (among five patients, three with ganciclovir and two with foscarnet) with ulcerative colitis. The eradication of CMV with antiviral drugs can improve the symptoms of severe and refractory ITP (DiMaggio et al., 2009). However, the apparent toxic effects of these drugs should also be considered. Neutropenia is the most common toxicity associated with ganciclovir (Venton et al., 2014), whereas foscarnet may cause kidney toxicity (Goldfarb and Coe, 1998). Renal toxicity is associated with cidofovir (Moyle, 2000). These drugs can cause significant adverse events, as well as long-term toxicity in children.

**FIGURE 3:**
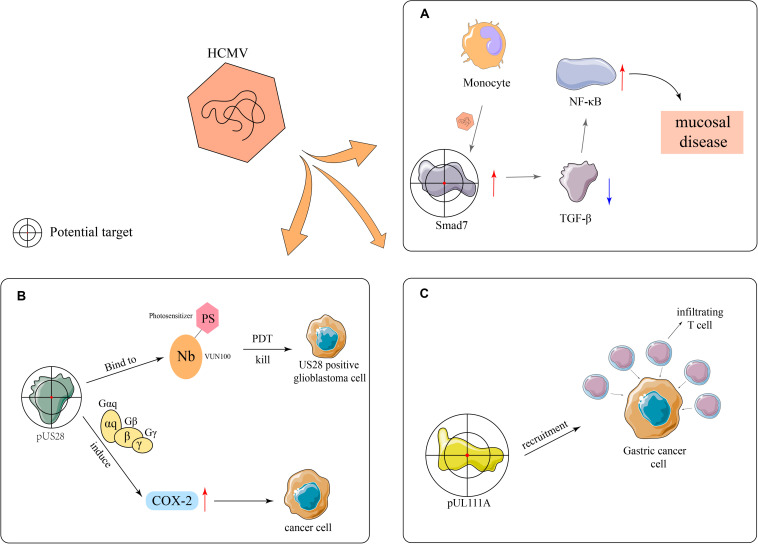
Potential therapeutic targets. **(A)** HCMV upregulates the expression of the TGF-β antagonist Smad7 in monocytes, thereby reducing the activation effects of TGF-β toward NF-κB and ultimately resulting in mucosal diseases. **(B)** VUN100 can bind the viral pUS28 during photodynamic therapy and can selectively kill US28-expressing glioblastoma cells. In addition, pUS28 promotes the expression of inflammatory factors, such as COX-2, to catalyze inflammatory processes through the activation of Gα_*q*_ and Gβγ and participates in tumor onset and development. **(C)** The overexpression of UL111A leads to the recruitment of infiltrating T cells in gastric cancer.

HCMV has the ability to upregulate the expression of Smad7, an intracellular antagonist of the transforming growth factor beta (TGF-β) signaling, in monocytes and attenuate the inhibitory effect of stromal TGF-β on NF-κB, thus promoting mucosal inflammation (Dennis et al., 2018; Figure 3A). In addition, Dennis et al. (2018) found that the expression of Smad7 antisense oligonucleotide in monocytes restores susceptibility to stromal TGF-β-induced inflammation anergy, which indicates that Smad7 may be used as a potential therapeutic target for CMV mucosal disease. VUN100, a kind of nanobody, consists of antibody fragments extracted from heavy-chain antibodies from the family Camelidae and can bind to the N-terminus and extracellular loop 3 (ECL3) of *US28.* It can selectively kill US28-expressing tumor cells during photodynamic therapy (De Groof et al., 2019). Additionally, *US28* can induce the expression of inflammatory proteins, such as cyclooxygenase-2 (COX-2), by activating Gαq and Gβγ, and subsequently the synthesis of PGE2, which promotes the onset of inflammation and initiates or aggravates tumor formation (Maussang et al., 2009). Specific COX-2 inhibitors can prevent HCMV replication and reduce the expression of *US28* in tumor cells, suggesting that HCMV plays an important role in medulloblastoma and US28 is a potential therapeutic target (Maussang et al., 2009; Baryawno et al., 2011; Figure 3B). The overexpression of the UL111A protein may result in the recruitment of multiple infiltrating T cells, thereby inhibiting the growth and invasion of gastric cancer cells. Therefore, *UL111A* may be another potential therapeutic target (Liu et al., 2020; Figure 3C).

Promising therapeutic targets aimed at the treatment of HCMV-related diseases have recently emerged, such as the viral terminase complex and the viral pUL97 kinase (Piret and Boivin, 2019). Currently, breakthrough anti-HCMV infection drugs based on these two targets include letermovir and maribavir (Britt and Prichard, 2018). Letermovir inhibits the formation of mature HCMV virus particles by targeting the pUL56 subunit of the viral terminase complex (Britt and Prichard, 2018) and has showed efficacy in a Phase III clinical trial. It has been approved for use in hematopoietic stem cell transplant recipients who are seropositive for HCMV as it can prevent the reactivation and re-infection of CMV (Piret and Boivin, 2019). Meanwhile, maribavir can influence the key processes involved in CMV replication, specifically the formation of viral envelopes and the discharge of viral particles, through the targeted inhibition of the protein kinase UL97 in CMV (Britt and Prichard, 2018). The drug showed good treatment efficacy in a Phase II trial and may potentially satisfy the treatment needs of patients with antiviral drug resistance. Maribavir is currently being tested in a Phase III trial, during which its efficacy will be further evaluated (Winston et al., 2008; Piret and Boivin, 2019). Studies on the treatment of HCMV infection have progressed relatively slowly and some of these aspects remain to be explored. The advent of letermovir and maribavir will promote the development of other antiviral drugs for the prevention and treatment of HCMV infection, thus improving the treatment and prognosis of HCMV-infected patients (Britt and Prichard, 2018).

## Conclusion

We have provided a summary of recent literature on *HCMV* genes involved in the viral replication cycle, immune evasion, and HCMV-related diseases. Further studies are needed to characterize the functions of multiple newly identified HCMV ORFs, such as *UL121* and *UL120*. To identify potential new targets for antiviral drug discovery and development, studies elucidating the molecular mechanisms by which *HCMV* genes contribute to disease development are warranted.

## Author Contributions

LY, GG, and XX planned the work. LY, YQ, and WY drafted the manuscript. YQ, WY, GG, HW, and XX revised the manuscript. LY, YQ, WY, GG, HW, and XX participated in the literature search and discussion. WY arranged figures. All authors read and approved the final manuscript.

## Conflict of Interest

The authors declare that the research was conducted in the absence of any commercial or financial relationships that could be construed as a potential conflict of interest.

## Abbreviations

AIDS: Acquired immunodeficiency syndrome
cGAMP: cyclic guanosine monophosphate-adenosine monophosphate
cGAS: cyclic guanosine adenosine synthase
COX-2: cyclooxygenase-2
CRC: colorectal cancer
Daxx: death domain associated protein
E: early
E/L: early-late
ECL3: extracellular loop 3
GC: gastric cancer
HCMV: human cytomegalovirus
HDACs: histone deacetylases
hDaxx: human death domain-associated protein 6
IE: immediate early
IFI16: interferon gamma-inducible protein 16
IFNs: type I interferons
IL-10: interleukin 10
ITP: idiopathic thrombocytopenic purpura
L: late
MIE: major immediate early
MITA: mediator of IRF3 activation
ND10: nuclear domain 10
NEC: nuclear egress complex
NF- κ B: nuclear factor kappa-B
NK: natural killer cells
NRG2: neuregulin-2
Nrp2: neuropilin-2
PAMPs: pathogen-associated molecular patterns
PDGFR α: platelet-derived growth factor receptor α
PKC: protein kinase C
PML: promyelocytic leukemia protein
PRRs: pattern recognition receptors
RFs: restriction factors
SLE: systemic lupus erythematosus
Sp100: 100-kDa speckled protein
SSc: systemic sclerosis
TGF β RIII: transforming growth factor β receptor type 3
TGF- β: transforming growth factor beta
vAC: virion assembly compartment.
